# Insights into *xanthomonas axonopodis* pv. *citri* biofilm through proteomics

**DOI:** 10.1186/1471-2180-13-186

**Published:** 2013-08-07

**Authors:** Tamara Zimaro, Ludivine Thomas, Claudius Marondedze, Betiana S Garavaglia, Chris Gehring, Jorgelina Ottado, Natalia Gottig

**Affiliations:** 1 Instituto de Biología Molecular y Celular de Rosario, Consejo Nacional de Investigaciones Científicas y Técnicas (IBR-CONICET), Ocampo y Esmeralda, Rosario, Santa Fe, Argentina; 2 Division of Biological and Environmental Sciences and Engineering, King Abdullah University of Science and Technology, Thuwal 23955-6900, Saudi Arabia

**Keywords:** *Xanthomonas axonopodis* pv. *citri*, Citrus canker, Biofilm, Proteomics

## Abstract

**Background:**

*Xanthomonas axonopodis* pv. *citri* (*X*. *a*. pv. *citri*) causes citrus canker that can result in defoliation and premature fruit drop with significant production losses worldwide. Biofilm formation is an important process in bacterial pathogens and several lines of evidence suggest that in *X*. *a*. pv. *citri* this process is a requirement to achieve maximal virulence since it has a major role in host interactions. In this study, proteomics was used to gain further insights into the functions of biofilms.

**Results:**

In order to identify differentially expressed proteins, a comparative proteomic study using 2D difference gel electrophoresis was carried out on *X*. *a*. pv. *citri* mature biofilm and planktonic cells. The biofilm proteome showed major variations in the composition of outer membrane proteins and receptor or transport proteins. Among them, several porins and TonB-dependent receptor were differentially regulated in the biofilm compared to the planktonic cells, indicating that these proteins may serve in maintaining specific membrane-associated functions including signaling and cellular homeostasis. In biofilms, UDP-glucose dehydrogenase with a major role in exopolysaccharide production and the non-fimbrial adhesin YapH involved in adherence were over-expressed, while a polynucleotide phosphorylase that was demonstrated to negatively control biofilm formation in *E*. *coli* was down-regulated. In addition, several proteins involved in protein synthesis, folding and stabilization were up-regulated in biofilms. Interestingly, some proteins related to energy production, such as ATP-synthase were down-regulated in biofilms. Moreover, a number of enzymes of the tricarboxylic acid cycle were differentially expressed. In addition, *X*. *a*. pv. *citri* biofilms also showed down-regulation of several antioxidant enzymes. The respective gene expression patterns of several identified proteins in both *X*. *a*. pv. *citri* mature biofilm and planktonic cells were evaluated by quantitative real-time PCR and shown to consistently correlate with those deduced from the proteomic study.

**Conclusions:**

Differentially expressed proteins are enriched in functional categories. Firstly, proteins that are down-regulated in *X*. *a*. pv. *citri* biofilms are enriched for the gene ontology (GO) terms ‘generation of precursor metabolites and energy’ and secondly, the biofilm proteome mainly changes in ‘outer membrane and receptor or transport’. We argue that the differentially expressed proteins have a critical role in maintaining a functional external structure as well as enabling appropriate flow of nutrients and signals specific to the biofilm lifestyle.

## Background

*Xanthomonas axonopodis* pv. *citri* (*X*. *a*. pv. *citri*) is a gram-negative plant pathogenic bacteria that causes citrus canker [1]. This phytopathogen invades host plant tissues entering through stomata or wounds and then colonizes the apoplast of fruits, foliage and young stems and symptoms of infection appear as raised corky lesions. At the final stage, plant tissue epidermis is broken due to cell hyperplasia, which allows bacterial dispersal to other plants by windblown rain. Persistent and severe disease can lead to defoliation, dieback and fruit drop thereby reducing yields, and hence causing serious economic losses. Moreover, quarantine restrictions are imposed to producing areas with citrus canker thus hampering commercialization of fresh fruit [2].

In most environments, bacteria primarily grow in association with surfaces, leading to the formation of biofilms. These biofilms generally consist of microbial cells attached to a surface and covered with an extracellular matrix composed of protein and polysaccharides [3]. The elevated population density forming a biofilm can increase biological processes that single cells cannot perform. Specifically, the biofilm lifestyle can offer increased protection against environmental stresses and increase bacterial resistance against host defense responses and antimicrobial tolerance. Biofilms also allow for consortial metabolism and may increase the possibility for horizontal gene transfer [3]. For most pathogenic bacteria, attachment to surfaces and successive biofilm formation are essential steps in the development of chronic infections and maintenance on host tissues [4]. In plant pathogens, biofilm formation also allows for increased bacterial cell density that in turn helps to achieve a critical mass of cells at a specific location to initiate and sustain interactions with host plants [5].

*X*. *a*. pv. *citri* biofilm formation appears to be a common feature during infection and different *X*. *a*. pv. *citri* mutants impaired in surface attachment, aggregation and hence in biofilm formation are also deficient in pathogenesis [6-8]. The lack of exopolysaccharide (EPS), the main component of the matrix surrounding biofilm cells, reduces epiphytic survival *in planta*[9] and has a negative impact on *X*. *a*. pv. *citri* virulence [10-14]. Other mutant strains affected in lipopolysaccharide (LPS) or glucan biosynthesis are impaired in the formation of structured biofilms and show reduced virulence symptoms [15-17]. Moreover, the two-component regulatory system ColR/ColS, which plays a major role in the regulation of *X*. *a*. pv. *citri* pathogenicity, also modulates biofilm formation [18]. In this context, further insight into *X*. *a*. pv. *citri* biofilm formation was gained by screening *X*. *a*. pv. *citri* transposon insertion mutants for biofilm-defective phenotypes, leading to the identification of several genes related to *X*. *a*. pv. *citri* biofilm formation [19]. Given that for *X*. *a*. pv. *citri* too, biofilm formation is a requirement to achieve maximal virulence, we have used proteomics to identify differentially expressed proteins with a view to gain further insight into the process of biofilm formation.

## Results and discussion

Phenotypic analysis of *X*. *a*. pv. *citri* biofilm development

**Figure 1:**
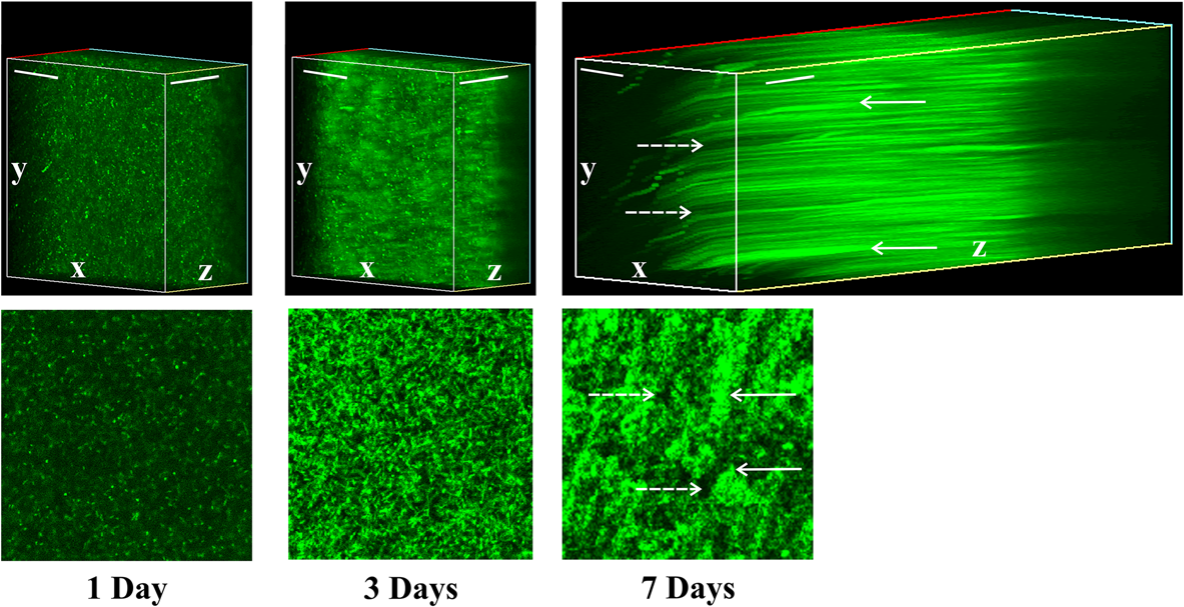
**Confocal laser scanning microscopy analysis*X. a*.pv.*citri in vitro*biofilms.** Representative photographs of laser scanning confocal analysis of GFP-expressing *X*. *a*. pv. *citri* cells cultured in static liquid XVM2 in 24-well PVC plates for one, three and seven days (upper panels). Serial images were taken at 0.5 μm distances (z-stack). White arrows point to cell aggregations and dotted white arrows point to network channels. Scale bars: 30 μm. For a better visualization, the lower panels are images of biofilm channels and cell aggregates at 7 days.

Biofilm formation generally requires a number of different processes including the initial surface attachment of cells, cell multiplication to form micro-colonies and maturation of the biofilm [20]. For a better understanding of the dynamics of this process in *X*. *a*. pv. *citri*, biofilm structure of a GFP-expressing *X*. *a*. pv. *citri* strain (Xac-GFP) was observed at different growth stages by confocal laser scanning microscopy. To this end, Xac-GFP was cultured in static liquid XVM2 medium, a minimal medium that mimics the nutritional conditions found in plant tissues [21]. As previously described, biofilms are important for *X*. *a*. pv. *citri* virulence, and thus XVM2 medium was used to analyze bacterial biofilm formation in a plant-like environment. After one day of growth, some cells began to attach to the surface of the PVC plate wells, however, the majority of cells remained dispersed in the culture medium (Figure 1). After three days of growth, cells initiated accumulation and formation of a biofilm (Figure 1), and after seven days, Xac-GFP cells formed a distinctly structured and dense biofilm consisting of large cell aggregations separated by a network of large channels (Figure 1) that ensured appropriate micronutrient and oxygen fluxes [22]. We also evaluated the population size of these biofilms and observed that at day seven of growth the biofilms reached a maximum population size of 1 x 10^9^ cfu/ml. In a planktonic culture in XVM2 medium, a similar maximal population size is reached in early stationary phase. Therefore, these two conditions of growth were used to identify differentially expressed proteins between the two lifestyles at their respective maximum population sizes and prior to the occurrence of noticeable cell death.

Two-dimensional gel electrophoretic analysis of protein expression and mass spectrometric identification of the *X*. *a*. pv. *citri* biofilm proteome

**Figure 2:**
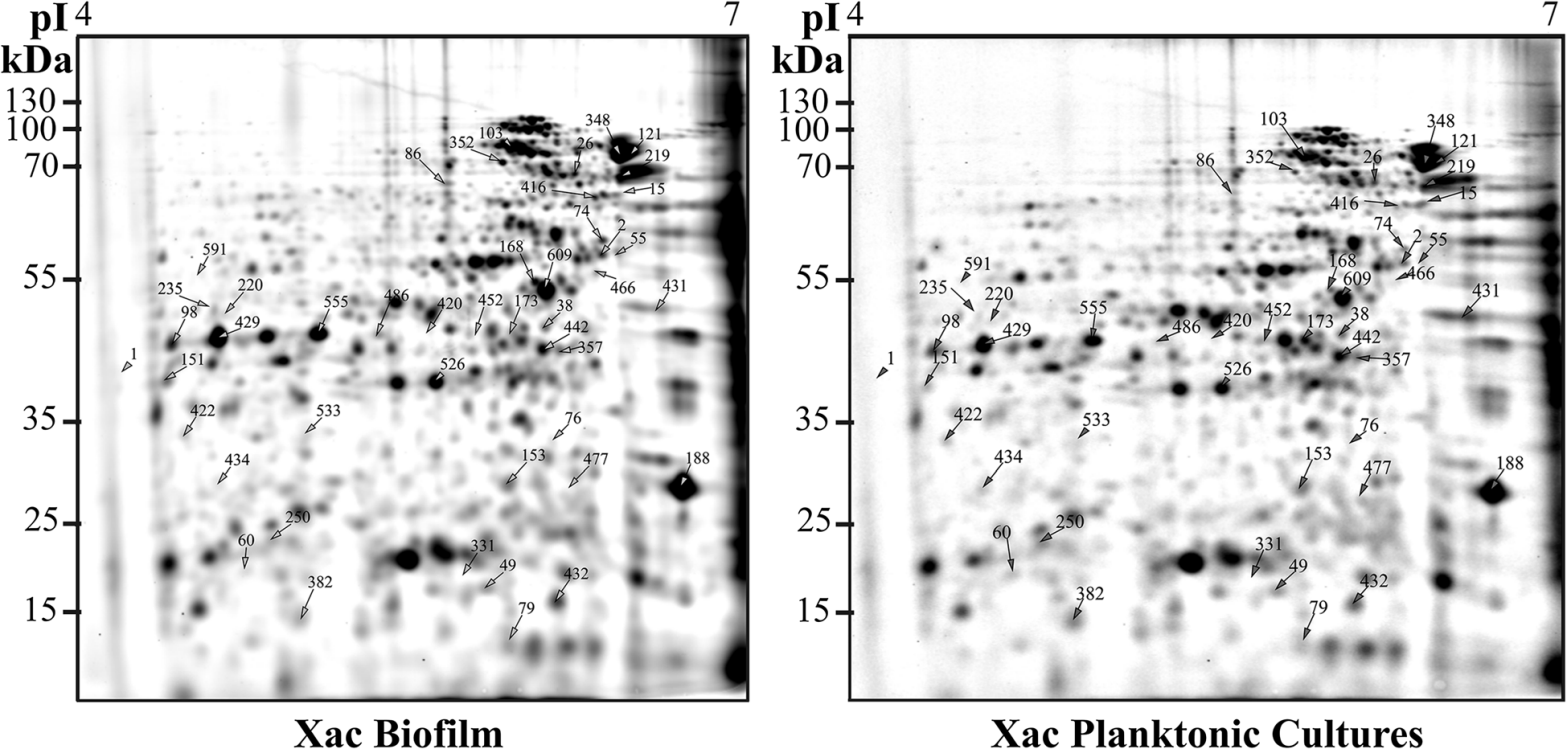
**Proteome profiles of *X*. *a*. pv. *citri *biofilms and planktonic cultures.** Proteins extracts (approximately 50 μg) from *X*. *a*. pv. *citri* biofilms (left gel) and planktonic cultures (right gel) were separated by 2D gel electrophoresis using 7-cm IPG strips pH range 4–7 and 12% SDS-PAGE. Proteome profiles of the cultures were compared using the Delta-2D (Decodon, Greifswald, Germany) analysis software.

Since proteomics is a powerful method to obtain systems information on the physiology of bacterial cells, we aimed at analyzing and characterizing mature biofilms of *X*. *a*. pv. *citri*, and compare the proteome to that of planktonic *X*. *a*. pv. *citri* cells. Total proteins of these cultures were extracted and separated by two-dimensional gel electrophoresis (2-DE) (see “Methods” section). Protein extractions were performed from three independent biological samples, and two technical replicate gels for each cell type were compared. A total of 46 protein spots were differentially regulated (Figure 2), excised and processed for analysis by mass spectrometry. Forty-one spots were identified, corresponding to a total of 53 proteins (Additional file 1: Table S1), while five spots (spots 250, 382, 348, 352 and 357) remained unidentified, probably due to the very low protein concentration in these spots. Some protein spots were assigned to more than one protein, possibly because the proteins co-migrated as a result of having the same p*I* and molecular weight. This pattern of co-migration is not uncommon in proteomic studies and was reported previously [23,24]. The 31 up- and 22 down-regulated *X*. *a*. pv. *citri* biofilm proteins were classified into different categories based on their functions [25] (Additional file 1: Table S1). The protein spot displaying the strongest up-regulation was 50S ribosomal protein L4 (XAC0973; +5.1 fold; spot 79), followed by TonB-dependent receptor (XAC3489; +4.9 fold; spot 168), while the protein spot with the most pronounced down-regulation was an ATP synthase beta chain (XAC3649; -10.7 fold; spot 76). Here we focus on interpreting a subset (see Table 1) of the differentially expressed biofilm proteins.

**Table 1:** **Selected proteins differentially expressed during *X***. ***a***. **pv**. ***citri *biofilm formation**

**Spot no.**	**Protein name**	**MOWSE score**	**Accession no.**	**Species**	**Gene ID in Xac**^**a**^	**Predicted MW/p*I***	**Observed MW/p*I***	**Peptide match/** **coverage**	**Fold change**^**b**^
01 Metabolism									
01.02 Nitrogen, sulfur and selenium metabolism									
01.02.02 Nitrogen metabolism									
60	NAD(PH) nitroreductase	111	Y587_XANC5	*X*. *c*. *pv*. *vesicatoria*	XAC0554	21.0/5.83	20.0/4.6	7/31%	−5.6
01.05 C-compounds and carbohydrate metabolism									
220	UDP-glucose dehydrogenase	125	Q8PGN5_XANAC	*X*. *a*. *pv*. *citri*	XAC3581	43.1/6.18	68.0/6.7	13/25%	+2.6
01.06 Lipid, fatty acid and isoprenoid metabolism									
01.06.02 Membrane lipid metabolism									
609	Outer membrane protein (FadL)	1070	Q8PRE4_XANAC	*X*. *a*. *pv*. *citri*	XAC0019	47.3/5.18	54.0/6.0	54/40%	+2.6
01.20 Secondary metabolism									
533	Coproporphyinogen-III oxidase, aerobic	191	HEM6_XANAC	*X*. *a*. *pv*. *citri*	XAC4109	34.6/5.81	48.0/5.4	11/30%	−1.5
434	Short chain dehydrogenase	141	Q8PME5_XANAC	*X*. *a*. *pv*. *citri*	XAC1484	26.0/5.97	29.0/4.5	14/34%	−5.1
02 Energy									
02.04 Glyoxylate cycle									
331	KDPG and KHG aldolase	163	Q8PKU5_XANAC	*X*. *a*. *pv*. *citri*	XAC2067	22.9/5.24	23.0/4.8	7/31%	−2.0
02.10 Tricarboxylic-acid pathway									
98	Malate dehydrogenase	905	MDH_XANAC	*X*. *a*. *pv*. *citri*	XAC1006	34.9/5.37	48.0/4.3	46/51%	+1.5
121	Dihydrolipoamide S-succinyltransferase	136	Q3BVA5_XANC5	*X*. *c*. *pv*. *vesicatoria*	XAC1534	42.4/5.87	69.0/6.5	9/10%	+1.8
235	Citrate synthase	218	Q3BPS8_XANC5	*X*. *c*. *pv*. *vesicatoria*	XAC3388	47.9/5.97	68.0/6.6	8/20%	+2.6
591	Succinate dehydrogenase flavoprotein subunit	206	Q3BTD_XANC5	*X*. *c*. *pv*. *vesicatoria*	XAC2077	65.8/5.89	55.0/4.4	18/22%	−7.4
02.45 Energy conversion and regeneration									
02.45.15 Energy generation									
76	ATP synthase beta chain	72	Q2P7Q4_XANOM	*X*. *o*. *pv*. *oryzae*	XAC3649	51.0/5.18	32.0/6.1	3/8%	−10.7
442	Phosphoglycerate kinase	688	AAM38190	*X*. *a*. *pv*. *citri*	XAC3347	40.9/4.91	45.0/6.0	47/43%	−1.9
422	NADH-ubiquinone oxidoreductase	40	Q3BRN4_XANC5	*X*. *c*. *pv*. *vesicatoria*	XAC2699	48.8/6.32	33.0/4.4	8/18%	−3.9
11 Transcription									
11.04 RNA processing									
153	Polynucleotide phosphorylase	137	PNP_XANAC	*X*. *a*. *pv*. *citri*	XAC2683	75.5/5.47	28.0/5.9	6/3%	−1.5
12 Protein synthesis									
12.01 Ribosome biogenesis									
79	50S ribosomal protein L4	133	AAM35856	*X*. *a*. *pv*. *citri*	XAC0973	21.8/9.68	14.0/5.9	4/15%	+5.1
12.04 Translation									
26	Elongation factor Tu	294	Q3BWY6_XANC5	*X*. *c*. *pv*. *vesicatoria*	XAC0957	43.3/5.45	67.0/6.2	25/24%	+2.2
173	Elongation factor Tu	329	Q3BWY6_XANC5	*X*. *c*. *pv*. *vesicatoria*	XAC0957	43.3/5.45	48.0/5.9	20/42%	+4.4
14 Protein fate (folding, modification and destination)									
14.01 Protein folding and stabilization									
416	Chaperone protein DnaK	98	DNAK_XANOM	*X*. *o*. *pv*. *oryzae*	XAC1522	68.9/5.02	66.0/6.3	10/12%	+2.9
20 Cellular transport, transport facilities and transport routes									
20.03 Transport facilities									
151	Regulator of pathogenicity factors	104	Q8PJM6_XANAC	*X*. *a*. *pv*. *citri*	XAC2504	41.3/5.98	41.0/4.3	8/21%	+3.2
429	Regulator of pathogenecity factors	729	Q8PJM6_XANAC	*X*. *a*. *pv*. *citri*	XAC2504	41.3/5.98	47.0/4.5	55/61%	+2.7
486	Regulator of pathogenecity factors	231	Q8PJM6_XANAC	*X*. *a*. *pv*. *citri*	XAC2504	41.3/5.98	48.0/5.2	16/30%	+2.2
526	*Regulator of pathogenecity factors	183	Q3BS50_XANC5	*X*. *c*. *pv*. *vesicatoria*	XAC2504	46.4/7.10	48.0/5.3	16/21%	+1.8
555	*Regulator of pathogenecity factors	148	Q3BS50_XANC5	*X*. *c*. *pv*. *vesicatoria*	XAC2504	46.4/7.10	42.0/4.9	11/12%	+2.8
30 Cellular communication/Signal transduction mechanism									
103	OmpA-related protein	371	Q8PER6_XANAC	*X*. *a*. *pv*. *citri*	XAC4274	110.1/5.29	75.0/5.9	28/16%	+2.9
1	TonB-dependent receptor	1406	Q8PI48_XANAC	*X*. *a*. *pv*. *citri*	XAC3050	105.8/4.76	42.0/4.1	89/34%	+2.9
2	TonB-dependent receptor	1441	Q8PI48_XANAC	*X*. *a*. *pv*. *citri*	XAC3050	105.8/4.76	58.0/6.7	85/35%	+2.9
74	TonB-dependent receptor	597	Q8PI48_XANAC	*X*. *a*. *pv*. *citri*	XAC3050	105.8/4.76	20.0/4.7	27/15%	+3.4
219	TonB-dependent receptor	356	Q8PI48_XANAC	*X*. *a*. *pv*. *citri*	XAC3050	105.8/4.76	68.0/6.4	23/23%	+2.2
466	TonB-dependent receptor-precursor	113	Q8PI27_XANAC	*X*. *a*. *pv*. *citri*	XAC3071	97.3/5.14	54.0/6.8	7/4%	+3.6
55	*TonB-dependent receptor	166	Q2HPF0_9XANT	*X*. *a*. *pv*. *glycines*	XAC3489	88.9/4.93	58.0/6.4	8/9%	+2.8
168	TonB-dependent receptor	636	Q8PGX3_XANAC	*X*. *a*. *pv*. *citri*	XAC3489	89.0/5.00	55.0/6.0	38/29%	+4.9
38	*TonB-dependent receptor	594	Q8PHT1_XANAC	*X*. *a*. *pv*. *citri*	XAC3168	87.3/5.20	48.0/6.0	44/21%	−1.8
15	TonB-dependent receptor	229	Q8PH16_XANAC	*X*. *a*. *pv*. *citri*	XAC3444	103.2/4.79	66.0/6.4	20/14%	−3.5
30.01.05.01 Protein kinase									
49	Adenylate kinase	93	Q3BPM9_XANC5	*X*. *c*. *pv*. *vesicatoria*	XAC3437	19.9/5.33	18.0/5.9	8/24%	−2.4
420	Histidine kinase- 2 component sensor system	40	Q3BTZ4_XANC5	*X*. *c*. *pv*. *vesicatoria*	XAC1991	45.9/5.33	48.0/5.5	10/13%	−2.2
34 Interaction with the environment									
86	YapH protein	51	Q8PKM0_XANAC	*X*. *a*. *pv*. *citri*	XAC2151	306.9/4.15	68.0/5.5	1/0%	+4.1
42 Biogenesis of cellular components									
42.27 Extracellular/secretion protein									
432	OmpW family outer memb. prot. precursor	151	Q3BP00_XANC5	*X*. *c*. *pv*. *vesicatoria*	XAC3664	23.8/4.97	17.0/6.1	5/13%	+2.2

^a^ Gene accession number in *X*. *axonopodis* pv. *citri* genome of the identified protein.

^b^ Fold change in biofilm compared to planktonic cultures.

* Protein spots 55 and 38 were previously identified as “outer membrane active sucrose transporter” and “ferric enterobactin receptor” are now classified as TonB-dependent receptor, while protein spots 526 and 555 were previously identified as “carbohydrate selective porin” and is now classified as Regulator of pathogenecity factors.

Functional characterization of differentially regulated *X*. *a*. pv. *citri* biofilm proteins

**Figure 3:**
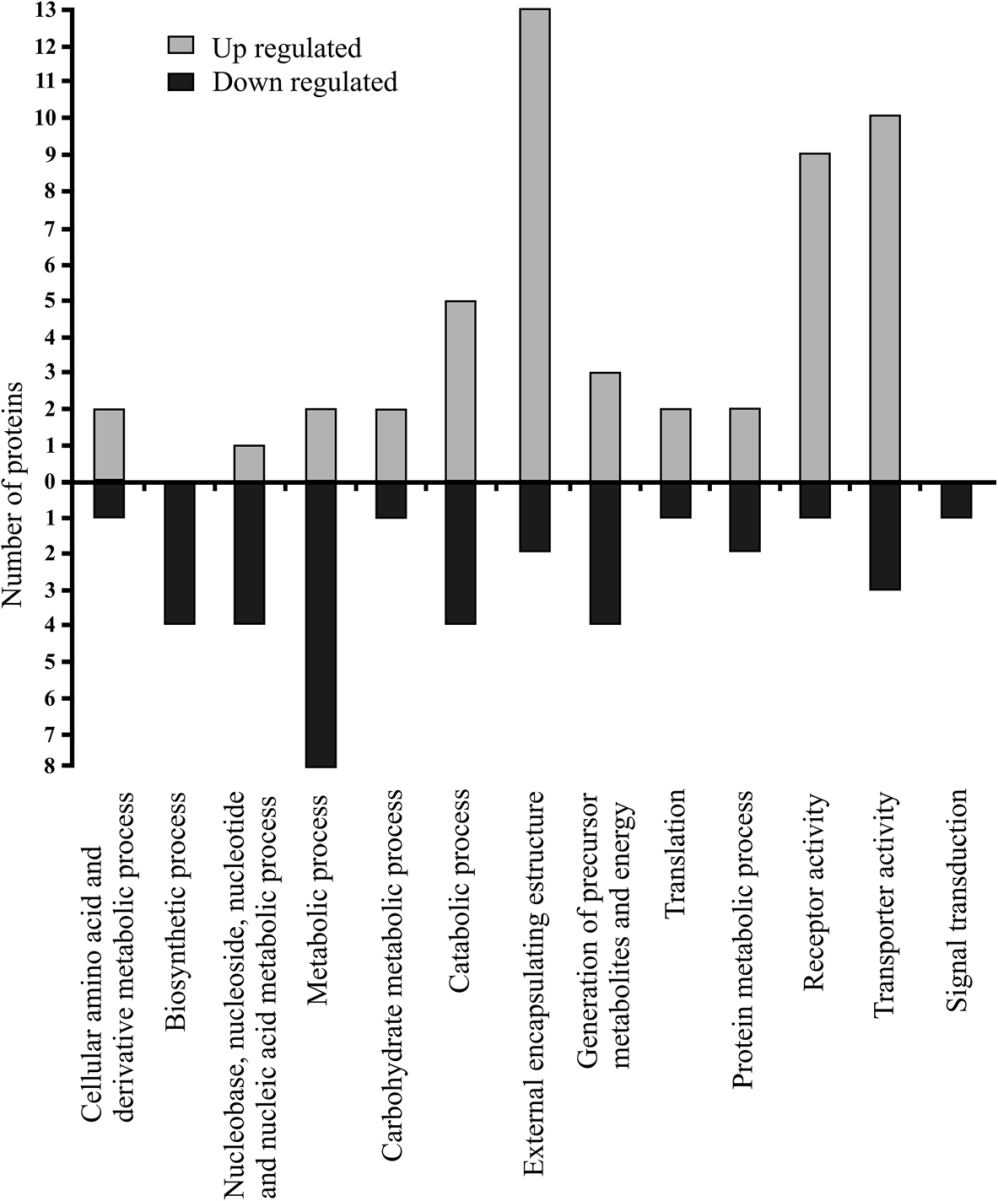
**Gene ontology (GO) terms enriched in the identified up-and down-regulated proteins in *X*. *a*. pv. *citri *biofilms compared to planktonic cultures.** Proteins were considered differentially expressed in *X*. *a*. pv. *citri* biofilms when variation was a minimum of 1.5-fold (p < 0.05). The GO enrichment analysis was performed using Blast2GO.

The identified differentially expressed proteins were used to determine enriched GO categories in biological processes, molecular function and cellular localization. The main enriched categories for the up- and down-regulated proteins with an average fold change of minimum ±1.5 are represented graphically (Figure 3). The major biological processes and cellular localization categories that changed in the *X*. *a*. pv. *citri* biofilms are ‘transporter activity’ and ‘external encapsulating structure’, respectively. The categories that showed enrichment in the up-regulated proteins include ‘catabolic process’, ‘external encapsulating structure’, ‘receptor activity’ and ‘transporter activity’; while most of the down-regulated proteins were in the categories of ‘biosynthetic process’, ‘nucleobase, nucleoside, nucleotide and nucleic acid metabolic process’, ‘metabolic process’, ‘catabolic process’ and ‘generation of precursor metabolites and energy’.

It is noteworthy that among the identified proteins, some have previously been shown to be involved in biofilm formation or regulation in other pathogenic bacteria. These include a the non-fimbrial adhesin, YapH [26], the FadL porin [27], citrate synthase [28], UDP-glucose dehydrogenase [19], the molecular chaperone DnaK [29-31], the elongation factor Ef-Tu [29,32], the polynucleotide phosphorylase [33] and a TonB-dependent receptor protein [19] (Table 2). These findings further validate our experimental results.

**Table 2:** Differentially expressed proteins detected previously in biofilms

**Protein**	**Species**	**Reference**
Non-fimbrial adhesion, YapH	*X*. *axonopodis* pv. *phaseoli*	26
Outer membrane protein, FadL	*P*. *fluorescens*	27
Citrate synthase	*B*. *cenocepacia*	28
UDP-glucose dehydrogenase	*X*. *axonopodis* pv. *citri*	19
Molecular chaperone DnaK	*S*. *pneumoniae*, *S*. *mutants*, *P*. *intermedia*	29, 30, 31
Elongation factor Ef-Tu	*S*. *pneumoniae*, *X*. *fastidiosa*	29, 32
Polynucleotide phosphorylase	*E*. *coli*	33
TonB-dependent receptor	*X*. *axonopodis* pv. *citri*	19

Specifically for *X*. *a*. pv. *citri*, we observed only a small overlap with recently published data that identified genes involved in biofilm formation by transposon mutagenesis [19]. The common proteins include UDP-glucose dehydrogenase and a TonB-dependent receptor proteins [19]. A possible explanation for this may be that transposon mutagenesis also identifies genes that are indirectly involved in biofilm formation, and additionally many of the identified genes may be required for the first stages of biofilm formation, such as adherence to the surface. Here, we focused on the proteins present in mature biofilms and for this reason many of the genes found in the genome-wide scale assay may be not differently expressed in this structure.

The most enriched categories for the up-regulated proteins in *X*. *a*. pv. *citri* biofilm are ‘external encapsulating structure’, ‘transporter activity’ and ‘receptor activity’, and include the outer membrane receptors termed TonB-dependent receptors (TBDRs). Among them, the OmpA-related protein (XAC4274, spot 103) and TonB-dependent receptors (XAC3050, spots 1, 2, 74, 219; XAC3071, spot 466 and XAC3489, spots 55 and 168) were up-regulated, while the TonB-dependent receptors (XAC3168, spot 38 and XAC3444, spot 15) were down-regulated in *X*. *a*. pv. *citri* biofilms. The TBDR proteins are localized in the outer membrane of gram-negative bacteria and their most prominent recognized role is the transport of iron-siderophore complexes and cobalamin into the periplasm [34]. Transport via TBDRs is an active process requiring energy that is provided by the inner membrane TonB-ExbB-ExbD protein complex [35]. Generally, expression of the genes encoding for these receptors is activated under conditions of iron starvation and repressed in the presence of iron by the ferric-uptake regulator (Fur) repressor [36]. Several genome sequences of gram-negative bacteria were examined to determine the number of TBDRs present in each genome, and it was demonstrated that only a number of these bacteria, among them the *Xanthomonas* species, have an over-representation of TBDRs [37]. Most of the analyzed bacteria with an elevated number of TBDRs share the ability to metabolize complex carbohydrates. Therefore, it was postulated that some Xanthomonas TBDRs might be involved in the transport of plant-derived molecules [37], and this hypothesis was confirmed with the characterization of two TBDRs from *Xanthomonas campestris* pv. *campestris* and *Caulobacter crescentus*, that transport sucrose and maltodextrins, respectively [37,38]. It was also suggested that other TBDRs might be involved in signal transduction processes [39]. Our proteomics results suggest that TBDRs participates in *X*. *a*. pv. *citri* biofilm formation and probably has a role in the adaptation to this lifestyle by modulating carbohydrate transport and utilization or signal perception and/or transduction. This hypothesis is supported by a recent study in *X*. *a*. pv. *citri* that showed that a transposon insertion mutant in a different TBDR (XAC0144) resulted in impaired in biofilm formation [19].

Other proteins that were up-regulated in biofilms and belonging to the categories ‘transporter activity’ and ‘receptor activity’ processes were identified as outer membrane proteins (OMPs) or porins. Porins are integral membrane β-barrel proteins and act as a selective barrier enabling the passage of nutrients, waste products, water and chemical signals through formed pores [40]. Within the class of porins, FadL (XAC0019, spot 609), a protein that allows the passage of fatty acids [41], was up-regulated in *X*. *a*. pv. *citri* biofilms, and was previously observed as important for bacterial virulence [14]. In *Pseudomonas fluorescens*, FadL has been reported in biofilms on abiotic surfaces, and it has been suggested that the long chain of fatty acids bound to FadL alter surface hydrophobicity and therefore adhesion characteristics [27]. Interestingly, the outer membrane porin termed “Regulator of pathogenicity factors” (RpfN) in the *X*. *a*. pv. *citri* genome (XAC2504, spots 151, 429, 486, 526, 555) was also up-regulated in the biofilms. This particular porin is encoded in a genomic region along with genes specialized in internalization of fructose and was suggested to play a role in carbohydrate transport [42], that in turn may be necessary for *X*. *a*. pv. *citri* adaptation to biofilm lifestyle. Moreover, the *Burkholderia pseudomallei* homolog to RpfN, named OprB, was shown to be important for optimal biofilm formation [43]. The OmpW (XAC3664; spot 432) was another up-regulated porin in *X*. *a*. pv. *citri* biofilms. It is involved in the transport of small hydrophilic molecules across the bacterial outer membrane [44]. Recent studies in *Salmonella typhimurium* suggest that this porin may have a role in the protection of bacteria against various forms of environmental stress by operating as efflux channel for toxic compounds [45]. We therefore hypothesize that OmpW may be involved in protecting *X*. *a*. pv. *citri* biofilms.

UDP-glucose dehydrogenase (UGD) (XAC3581, spot 220) was over-expressed in *X*. *a*. pv. *citri* biofilms (Table 1) and enriched in the category ‘metabolic process’. This enzyme catalyzes the conversion of UDP-glucose to UDP-glucuronic acid and the cellular functions of UGD have been investigated in a number of organisms establishing a role in detoxification, polysaccharide biosynthesis as well as embryonic development [46]. Moreover, a double mutant in *Pseudomonas aeruginosa* UGD (PA2022-PA3559) produced thinner biofilms than the wild type PAO1 and it has been suggested that the functional role of UGD in *P*. *aeruginosa*, involves hyaluronic acid (polysaccharide consisting of alternative GlcUA and GlcNAc residues) synthesis, which also contributes to biofilm formation [47]. In *X*. *campestris* pv. *campestris*, UGD is required for the biosynthesis of xanthan gum that is a component of the exopolysaccharide (EPS). Therefore, together with the well established role of *X*. *a*. pv. *citri* EPS in bacterial adherence and biofilm formation [10,11,19], the over-expression of UGD in *X*. *a*. pv. *citri* biofilms is consistent with a major role of EPS under biofilm growth conditions. Also consistent with this conclusion is the absence of biofilm formation in a *X*. *a*. pv. *citri* UGD deletion mutant [19].

The non-fimbrial adhesin, YapH (XAC2151, spot 86), a protein up-regulated in *X*. *a*. pv. *citri* biofilms, is an adhesin that belongs to the family of the filamentous hemagglutinins secreted by the two-partner secretion system [48]. In *X*. *axonopodis* pv. *phaseoli*, a YapH ortholog was discovered to be involved in the adhesion process to biotic and abiotic surfaces and also in biofilm formation [26]. We previously characterized another filamentous hemagglutinin named *X*. *a*. pv. *citri* FhaB, and showed that it is critical for *X*. *a*. pv. *citri* biofilm formation [6]. In agreement with these studies, the present results substantiate the role of this family of adhesins in *X*. *a*. pv. *citri* biofilm formation.

Among the category ‘nucleic acid metabolic process’, the polynucleotide phosphorylase (PNPase) (XAC2683, spot 153) was down-regulated in biofilms. PNPase is an important enzyme involved in RNA processing and turnover [49]. Recently, it was demonstrated that PNPase negatively regulates cell aggregation and biofilm formation in *E*. *coli* by inhibiting the expression of genes involved in the production of the EPS poly-*N*-acetylglucosamine at post-transcriptional level [33]. In this context, our results may suggest that in *X*. *a*. pv. *citri*, this enzyme also enables the adaptation to the biofilm lifestyle.

Several proteins involved in other categories such as protein synthesis, folding and stabilization were up-regulated in *X*. *a*. pv. *citri* biofilm, including the Elongation factor Tu (Ef-Tu) (XAC0957, spots 26, 173), the 50s ribosomal protein L4 (XAC0973; spot 79) and the molecular chaperone DnaK (XAC1522, spot 416). Our results are in agreement with reports which described an increase in 30S ribosomal protein S1, Ef-Tu, 50s ribosomal protein L1, and DnaK during biofilm formation in *Streptococcus pneumoniae*[29]. Similarly, *Pseudomonas aeruginosa* biofilms display an up-regulation of ribosome recycling factor and 50S ribosomal protein [50]. The increase in Ef-Tu and the 50s ribosomal protein L4 observed in *X*. *a*. pv. *citri* biofilm may be related to participation in protein synthesis and folding and this in turn may be a specific requirements of the lifestyle. However, for Ef-Tu, other functions such as participation in bacterial aggregation also need to be considered since this factor has also been identified as a cell wall associated component in several bacterial species where it mediates the binding to host proteins (e.g. to human plasminogen in *Listeria monocytogenes*) [51] and in *Mycoplasma pneumoniae* where it directly binds fibronectin, suggesting a role of Ef-Tu in aggregation [52]. Ef-Tu was also over-expressed in the wild type strain of *Lactobacillus crispatus* as compared to an isogenic mutant that lost the aggregative phenotype and strengthening the claim for a role in adhesion [53]. Moreover, in the citrus pathogen *Xylella fastidiosa*, Ef-Tu was reported to be up-regulated in biofilms [32]. Recent work demonstrated that in *X*. *a*. pv. *citri*, DnaK is necessary for the bacteria to achieve full virulence [14]. Several proteomics reports associate the up-regulation of DnaK to biofilm formation. Among them, a *dnaK* knock-down mutant of *Streptococcus mutans* with reduced levels of DnaK (<95%) shows impaired biofilm-forming capacity [30], while DnaK expression was up-regulated in a *Prevotella intermedia* biofilm-forming strain when compared to a variant lacking biofilm formation [31].

Several proteins that were enriched in the categories ‘metabolic process’, ‘generation of precursor metabolites and energy’, ‘catabolic process’ and ‘biosynthetic process’ showed altered expression patterns in *X*. *a*. pv. *citri* biofilms. A number of enzymes of the tricarboxylic acid (TCA) cycle were also detected as differentially expressed in the biofilm compared to planktonic cultures. Since the TCA cycle plays a central role in metabolism, our finding indicates that the two lifestyles may have markedly different metabolic and energy requirements. The three differentially expressed enzymes of the TCA cycle are citrate synthase (XAC3388, spot 235), malate dehydrogenase (XAC1006, spot 98) and dihydrolipoamide S-succinyltransferase (XAC1534, spot 121). Citrate synthase catalyzes the first reaction in the TCA cycle converting oxaloacetate and acetyl-coenzyme A into citrate and coenzyme A (CoA). Incidentally, it has been observed that a citrate synthase of *Burkholderia cenocepacia* is necessary for optimum levels of biofilm formation and virulence [28]. In *Geobacter sulfurreducens*, uniform expression of citrate synthase genes was noted throughout biofilms [54]. The second over-expressed protein in biofilms was identified as malate dehydrogenase, the enzyme that catalyzes the reversible conversion of L-malate to oxaloacetate, and the synthesis of this enzyme is influenced by cell growth conditions such as oxygenation and the nature of carbon substrates [55]. Succinate dehydrogenase (spot 591) was down-regulated in the biofilm. Succinate dehydrogenase complex catalyzes the oxidation of succinate to fumarate, donating FADH_2_ for oxidative phosphorylation. In the presence of oxygen, the TCA cycle operates as an oxidative pathway coupled to aerobic respiration. Under oxygen-limiting conditions, the TCA cycle operates as reductive (incomplete) pathway dedicated largely to the synthesis of precursors blocking the steps from α-ketoglutarate to succinyl-CoA. In the reductive pathway, succinyl-CoA is synthesized using the enzyme fumarate reductase as an alternative to succinate dehydrogenase, leading to the down-regulation of succinate dehydrogenase [56]. Given the down-regulation of succinate dehydrogenase in *X*. *a*. pv. *citri* biofilms, our results might suggest that in biofilms, the TCA cycle is converted into the reductive pathway possibly because of oxygen limitations under these growth conditions. Accordingly, succinate dehydrogenase is directly linked to the respiratory chain and in *E*. *coli*, an increase in oxygen respiration correlated with succinate dehydrogenase over-expression [57]. Moreover, *X*. *a*. pv. *citri* biofilms showed a down-regulation of protein involved in energy generation, such as ATP-synthase (XAC3649, spot 76), phosphoglycerate kinase (XAC3347, spot 442) and NADH-ubiquinone oxidoreductase (XAC2699, spot 422). Phosphoglycerate kinase enzyme is involved in later reactions of the glycolytic pathway; therefore its inhibition should lead to an increased pool of glycolytic intermediates in the early steps that might benefit biosynthetic processes. Furthermore, an enzyme involved in cellular energy homeostasis, adenylate kinase (XAC3437, spot 49) was also down-regulated in *X*. *a*. pv. *citri* biofilms. This enzyme catalyzes the interconversion of adenine nucleotides generating ATP and AMP, a metabolic signaling molecule.

Several antioxidant enzymes enriched in the ‘metabolic process’ are involved in secondary metabolism. In bacteria, the normal course of aerobic metabolism produces reactive oxygen species (ROS) with the concomitant requirement for the constitutive expression of ROS scavenging systems such as antioxidant enzymes [58]. In accordance with our hypothesis that *X*. *a*. pv. *citri* biofilms have a reduced aerobic respiration rate, these biofilms showed a down-regulation in antioxidant enzymes like a NAD(PH) nitroreductase (XAC0554, spot 60), a short chain dehydrogenase (XAC1484, spot 434) and an aerobic coproporphyinogen-III oxidase (XAC4109, spot 533). Nitroreductases are a family of evolutionarily related proteins involved in the reduction of nitrogen-containing compounds and the short-chain dehydrogenases/reductases represent a large family of enzymes, most of which are NAD- or NADP-dependent oxidoreductases [59]. Coproporphyrinogen III oxidase is encoded by the *hemF* gene that is involved in heme-biosynthesis [60]. This protein was shown to be a member of the hydrogen peroxide (oxidative stress)-induced regulon responsible for protecting cells from oxidative damage [61]. The 4-hydroxy-2-oxoglutarate (KHG)/phospho-2-dehydro-3-deoxygluconate (KDPG) aldolases (XAC2067, spot 331), a key enzyme for the Entner-Doudoroff pathway, was down-regulated in biofilms. The KHG aldolase catalyzes the interconversion of 4-hydroxy-2-oxoglutarate into pyruvate and glyoxylate in the glyoxylate cycle, while KDPG-aldolase induces the interconversion of 6-phospho-2-dehydro-3-deoxy-D-gluconate into pyruvate and glyceraldehyde 3-phosphate and the two enzymes are structurally and functionally related [62]. One primary function of the glyoxylate cycle is to replenish the tricarboxylic and dicarboxylic acid intermediates that are normally provided by the TCA cycle. In *X*. *a*. pv. *citri* biofilms, several enzymes of the TCA cycle are up-regulated suggesting a reduced requirement for the glyoxylate cycle under this static growth condition.

One GO category (‘signal transduction’) is enriched in down-regulated proteins only and comprises a putative two-component system sensor histidine kinase under-expressed in *X*. *a*. pv. *citri* biofilms (XAC1991, spot 420). Previously, it was shown that a *X*. *a*. pv. *citri* mutant that has a transposon insertion at the intergenic region between XAC1990 and XAC1991 induces milder infection symptoms than the wild type strain [14]. Since these genes have the same genomic orientation, this mutation probably impairs only XAC1991 expression. These data may suggest that besides its involvement in *X*. *a*. pv. *citri* pathogenicity, this sensor histidine kinase may also be involved in the adaptation to different lifestyles.

### Transcriptional analysis of selected genes encoding differentially expressed proteins

**Figure 4:**
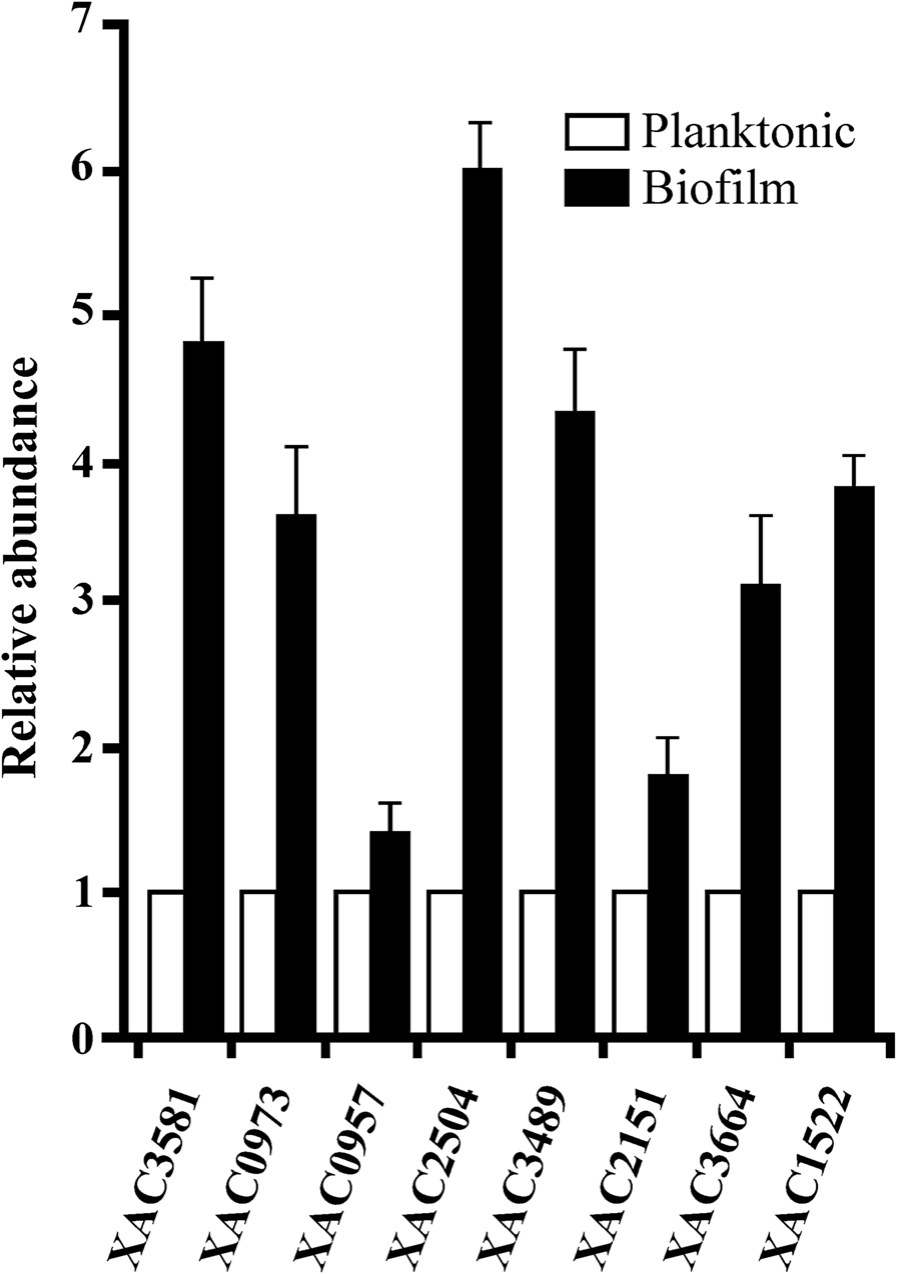
**Analysis of the expression of selected genes encoding differentially expressed proteins.** A significant difference in expression was detected by qRT-PCR between planktonic and biofilm conditions for selected genes confirming their expression during *X*. *a*. pv. *citri* biofilm formation. Black bars indicate the expression levels of *X*. *a*. pv. *citri* transcripts in biofilm compared to a reference planktonic growth (white bars). As a reference gene, a fragment of 16S rRNA was amplified. Values represent the means of four independent experiments. Error bars indicate standard deviations. Data were statistically analyzed using one-way ANOVA (p < 0.05) and Student *t*-test (p < 0.05).

We selected some of these genes for further validation by quantitative real-time PCR (qRT-PCR). Total RNA was extracted from *X*. *a*. pv. *citri* mature biofilms and from planktonic cells, both grown as for the proteomic study. Bacterial cDNA was obtained from 1 μg of total RNA in both growth conditions. The assay was performed with specific primers for the following *X*. *a*. pv. *citri* genes: XAC3581 (UDP-glucose dehydrogenase), XAC0973 (50S ribosomal protein L4), XAC0957 (EfTu), XAC2504 (RpfN), XAC3489 (TonB-dependent receptor), XAC2151 (YapH), XAC3664 (OmpW) and XAC1522 (DnaK). We noted that the changes in transcript levels of theses genes mirrored the changes observed in the proteomics analysis (p < 0.05) (Figure 4).

## Conclusions

Several lines of evidence indicate that *X*. *a*. pv. *citri* biofilm formation plays an important part in bacterial pathogenicity. Among them, studies on a variety of impaired biofilm forming mutants have revealed the importance of this lifestyle for the citrus pathogen. Here we identified proteins differentially expressed in a mature *X*. *a*. pv. *citri* biofilm as compared to free planktonic cultured cells. A larger number of differentially expressed proteins are down-regulated and relate to metabolic processes suggesting a reduction in energy requirements. Further experiments will show to which extent this is due to reduced metabolic activity at this growth stage. It is also noteworthy, firstly, that proteins that are down-regulated in *X*. *a*. pv. *citri* biofilms are enriched for the GO terms ‘generation of precursor metabolites and energy’ and secondly, that the biofilm proteome mainly displayed changes in outer membrane and receptor or transport proteins suggesting that they may have a role in maintaining a functional external structure as well as enabling appropriate flow of molecules and signals required in this lifestyle. This study is the first report of a *X*. *a*. pv. *citri* biofilm proteome and the information gained will support future comparative analyses of differentially expressed genes and/or proteins involved in biofilm formation. In addition, the data will also inform approaches to a more detailed physiological investigation into the function of individual proteins and their role in biofilm formation.

## Methods

### Bacterial strains, culture conditions and media

*X*. *axonopodis* pv. *citri* was grown at 28°C in Silva Buddenhagen (SB) medium (5 g/l sucrose, 5 g/lyeast extract, 5 g/l peptone, and 1 g/l glutamic acid, pH 7.0) and XVM2 medium (20 mM NaCl, 10 mM (NH_4_)_2_SO_4_, 1mM CaCl_2_, 0.01 mM FeSO_4_, 5 mM MgSO_4_, 0.16 mM KH_2_PO_4_, 0.32 mM K_2_HPO_4_, 10 mM fructose, 10 mM sucrose and 0.03% (w/v) casein acid hydrolysate (casaminoacid), pH 6.7). Bacteria were grown in SB with shaking until exponential growth phase and then diluted 1:10 in fresh XVM2 medium. For planktonic cultures these dilutions were grown under agitation at 200 rpm on a rotating shaker and cells were recovered after 24 hours of growth at early stationary phase. For biofilms, 2 ml-aliquot of these dilutions were placed in 24-well PVC plates and incubated statically for seven days at 28°C. In both cases the population size was estimated by recovering bacteria by centrifugation and plating adequate dilutions on SB plates. After 48 hours colonies were counted and related to the volume of the original cultures. The *X*. *axonopodis* pv. *citri* strain used in this work is named Xcc99-1330 and was kindly provided by Blanca I. Canteros (INTA Bella Vista, Argentina).

### Confocal analysis of biofilm architecture

The GFP-expressing *X*. *a*. pv. *citri* strain previously constructed using the parental strain Xcc99-1330 [6] was used in the present study and statically grown in 24-well PVC plates, as described above, and biofilm development was analyzed at 1, 3 and 7 days by confocal laser scanning microscopy (Nikon Eclipse TE-2000-E2).

### Protein extraction and resolubilization for the proteomic analysis

Cellular pellets of *X*. *a*. pv. *citri* planktonic and biofilm cultures were obtained by centrifugation and resuspended in urea buffer (9 M urea, 2 M thiourea and 4% (w/v) 3-[(3-cholamidopropyl)dimethylammonio]-1-propanesulfonate (CHAPS)) with vigorous vortexing at room temperature. Concentration of total protein extracts was estimated by Bradford assay using the Quick Start Bradford protein assay (Bio-Rad). Protein extracts were prepared from three different flasks for both growth conditions.

### CyDye labeling

Prior to 2D-PAGE, protein samples were labeled using the fluorescent cyanine three-dye strategy (CyDyes; GE Healthcare, Sweden), according to manufacturer’s instructions. Briefly, proteins (50 μg) of an internal standard containing an equal amount of the control and treated samples were incubated with 400 pmol of Cy2, freshly dissolved in dimethyl formamide (DMF), while *X*. *a*. pv. *citri* planktonic and *X*. *a*. pv. *citri* forming biofilm samples were labeled with Cy3 and Cy5, respectively. Dye swap between samples was carried out to avoid artifacts due to preferential labeling. Three biological replicates and two technical replicates were carried out, giving rise to a total of six gel images per growth conditions. All reactions were carried out on ice and in the dark to limit signal quenching. Labeling was performed for 30 min and terminated by incubation with 10 nmol lysine for 10 min. Equal volumes of urea lysis buffer containing 20 mg/ml DTT and 2% (v/v) IPG buffer, pH range 4–7 (GE Healthcare) were added to each sample and incubated for 15 min. After pooling the samples, the volume was adjusted to 125 μl with rehydration buffer (7 M urea, 2 M thiourea, 4% (w/v) CHAPS, 2 mg/ml DTT and 1% (v/v) IPG buffer pH 4–7, GE Healthcare) and separated by 2D-DIGE.

### Protein separation and quantification by 2D-DIGE electrophoresis

Labeled protein samples in urea lysis buffer were used to rehydrate 7 cm-long linear IPG strips, pH range 4–7 (GE Healthcare). Following overnight rehydration at room temperature, strips were focused for a total of 8,750 Vhrs 50 μA at 20°C, as follows: step, 500 V for 250 Vhrs; step, 1,000 V for 500 Vhrs and step, 8,000 V for 8,000 Vhrs. Prior to SDS-PAGE, strips were equilibrated twice for 15 min in equilibration buffer (50 mM Tris, pH 8.8, 30% (v/v) glycerol, 6 M urea, 2% (w/v) SDS) first containing 1% (w/v) DTT and then 2.5% (w/v) iodoacetamide with gentle shaking. Strips were loaded on top of 12% SDS-PAGE. Strips were sealed on top of the gel with 1% (w/v) agarose in SDS running buffer (25 mM Tris, 192 mM glycine, 0.1% (w/v) SDS). Gels were run at 50 V for the first 15 min and then at 100 V until the dye reached the bottom of the gels.

### Comparative analysis and protein identification

Gel images were obtained using the Typhoon^TM^ 9410 scanner (GE Healthcare). Cy2-labeled pool samples were imaged using a 488 nm blue laser and a 520 nm band-pass (BP) 40 emission filter. Cy3 images were obtained using a 532 nm green laser and a 520 nm BP30 emission filter, and the Cy5 images using a 633 nm red laser and a 670 nm BP30 emission filter. Images were analyzed with the Delta2D (Decodon, Greifswald, Germany) software. Spot quantities were calculated by summing pixel intensities within the spot boundaries and used for analyzing gene expression. Normalized expression profile data were used to statistically assess changes in protein spot expression. Differentially expressed protein spots between the two groups were calculated using the Student-T test with a critical p-value ≤ 0.05 and the permutation-based method to avoid biased results that may arise within replicate gels if spot quantities are not normally distributed. The adjusted Bonferroni correction was applied for false discovery rate (FDR) to control the proportion of false positives in the result set. Principal component analysis was performed to determine samples/spots that contributed most to the variance and their relatedness.

Differentially expressed protein spots of interest were manually excised and each placed into separate microcentrifuge tubes. Gel pieces were rinsed briefly with 100 μl of 25 mM NH_4_HCO_3_, incubated in 100 μl of 25 mM NH_4_HCO_3_ in 50% (v/v) acetonitrile (ACN) for 30 min with gentle shaking, dehydrated with 100 μl of 100% (v/v) ACN for 10 min and then rehydrated with 100 μl of 25 mM NH_4_HCO_3_ for 30 min with gentle shaking. Gel pieces were dehydrated again with 100 μl of 100% (v/v) ACN for 10 min and completely evaporated. Proteins were reduced with 50 μl of 10 mM DTT in 100 mM NH_4_HCO_3_ at 56°C for 45 min and then alkylated with 50 μl of 50 mM iodoacetamide in 100 mM NH_4_HCO_3_ for 30 min at room temperature in the dark. Gel pieces were rinsed with 200 μl of 100 mM NH_4_HCO_3_ and then with 200 μl of 100% (v/v) ACN for 10 min each step. These steps were repeated once more. Gel pieces were completely dehydrated and incubated with 200 ng of trypsin (Worthington Biochemical Corp., Lakewood, NJ) diluted in 50 mM NH_4_HCO_3_ overnight at 30°C. Samples were cooled down to room temperature and incubated with 20 μl of 20 mM NH_4_HCO_3_ for 10 min. Peptides were extracted twice from the gel pieces with 20 μl of 5% (v/v) formic acid (FA) in 50% (v/v) ACN for 10 min each, collected to separate tubes, evaporated and stored at −20°C prior to mass spectrometry analysis.

Digested peptide mixtures were suspended in 0.1% (v/v) formic acid (FA) in 5% (v/v) ACN, and analyzed with an LTQ Orbitrap mass spectrometer (Thermo Scientific, Bremen, Germany) equipped with an electrospray ion source and coupled to an EASY-nanoLC (Proxeon Biosystems, Odense, Denmark) for nano-LC-MS/MS analyses. A volume of 5 μl of the peptide mixture was injected onto a 5 μm, 300 Å, 50 mm long × 0.3 mm Magic C18AQ (Michrom, Thermo-Scientific) pre-column and a 3 μm, 100 Å, 100 mm long × 0.1 mm Magic C18AQ (Michrom, Thermo-Scientific) column. A spray voltage of 1,500 V was applied. The mobile phases consisted of 0.1% FA and 5% ACN (A) and 0.1% FA and 90% ACN (B). A three step gradient of 0-40% B in 20 min, then 40-90% B in 5 min and finally 90% B for 20 min with a flow of 500 nl/min over 45 min was applied for peptide elution. The MS scan range was *m*/*z* 350 to 1,600. The top 10 precursor ions were selected in the MS scan by Orbitrap with resolution r = 60,000 for fragmentation in the linear ion trap using collision-induced dissociation. The normalized collision-induced dissociation was set to 35.0.

All spectra were converted to mgf using Proteome Discover version 1.2 (Thermo-Scientific) and submitted to a local MASCOT (Matrix Science, London, UK) server and searched against bacteria in the SwissProt (release 57.15) and MSDB databases (release 9.0) with a precursor mass tolerance of 10 ppm, a fragment ion mass tolerance of 0.6 Da and strict trypsin specificity allowing up to one missed cleavage, carbamidomethyl as fixed modification and oxidation of methionine residues as variable modification. Proteins were considered positive if the MASCOT score was over the 95% confidence limit corresponding to a score > 35 for proteobacteria.

### RNA preparation and quantitative real-time PCR (qRT-PCR)

Total RNA from *X*. *a*. pv. *citri* mature biofilms and planktonic cells was extracted using TRIzol® reagent (Invitrogen), according to the manufacturer’s instructions. After DNAse (Promega) treatment, cDNA was synthesized from 1 μg of total RNA using M-MLV RT (Promega) and the oligonucleotide dN6 was added as follows: 200 U of M-MLV RT (Promega, USA), 0.25 μg of primer dN6 and 0.5 mM of deoxynucleoside triphosphates (dNTPs) (reaction final volume: 20 μl) and incubated for 1 h at 42°C, and then for 10 min at 94°C. The qRT-PCRs were performed by combining 1 μl of cDNA template, 0.5 U of Go Taq DNA polymerase (Promega), 1 × reaction buffer, 0.2 mM dNTPs and 20 pmol of each primer (final reaction volume, 20 μl) in a Mastercycler ep *realplex* thermal cycler (Eppendorf) using SYBR Green I (Roche) to monitor double-stranded DNA (dsDNA) synthesis. The qRT-PCR conditions were set to 95°C for 1 min, followed by 40 cycles of 95°C for 15 s, 55°C for 30 s and 72°C for 40 s. The primer pairs used for qRT-PCR are provided in Additional file 2: Table S2. As a reference gene, a fragment of 16S rRNA was amplified using the same qRT-PCR conditions. Values were normalized by the internal reference (Ct_r_) according to the equation ΔCt = Ct – Ct_r_, and quantified as 2^–ΔCt^. A second normalization using a control (time=0 days) (Ct_c_), ΔΔCt = Ct – Ct_c_, produces a relative quantification: 2^–ΔΔCt^[63]. Values are the means of four independent experiments. Results were analyzed using one-way ANOVA (p < 0.05) and Student *t*-test (p < 0.05).

### GO enrichment analysis

Proteins were considered as differentially expressed when variations between planktonic and biofilm grown cells were at least 1.5-fold and the quantitation p-value of 0.05. The GO enrichment analysis was performed using Blast2GO [64-66].

## Competing interests

The authors declare that they have no competing interests.

## Authors’ contributions

TZ, JO and NG conceived the project and designed the experiments. TZ, LT, CM and BSG designed and performed the experiments. All authors contributed to the analysis and interpretation of the data and LT, CM, BSG, CG, JO and NG wrote the manuscript. All authors read and approved the manuscript.

## Supplementary Material

Additional file 1: Table S1 Complete list of the differentially expressed proteins during *X*. *a*. pv. *citri* biofilm formation. Click here for file

Additional file 2: Table S2 Oligonucleotides used in qRT-PCR of selected genes. Click here for file
