# Supplementary material for: Insights into xanthomonas axonopodis pv. citri biofilm through proteomics
Source: BMC Microbiol. 2013 Aug 7;13:186. doi: 10.1186/1471-2180-13-186 (PMC3750573; doi:10.1186/1471-2180-13-186)
Supplement: Additional file 1: Table S1 — Complete list of the differentially expressed proteins during X. a. pv. citri biofilm formation. [file 1471-2180-13-186-S1.doc]

**Supplementary Table 1. Complete list of the differentially expressed proteins during *X. a.* pv. *citri*** biofilm formation

| **Spot no.** | **Protein name** | **MOWSE score** | **Accession no.** |  | **Predicted MW/p*I*** | **Observed MW/p*I*** | **Peptide match/**  **coverage** | **Fold change^a^** | **Biofilm samples^b^** | **Planktonic samples^c^** |
| --- | --- | --- | --- | --- | --- | --- | --- | --- | --- | --- |
| 01 Metabolism | | | | | | | | |  |  |
| 01.01 Amino acid metabolism | | | | | | | | |  |  |
| 98b | Ketol-acid reductoisomerase | 92 | ILVC_XANAC |  | 36.1/5.27 | 48.0/4.4 | 8/27% | +1.5 | 89.1±4.6 | 61.1±15.8 |
| 431 | N-acetylornithine carbamoyltransferase | 145 | AOTC_XANAC |  | 38.5/6.13 | 50.0/6.6 | 5/8% | -7.6 | 37.1±5.1 | 281.1±34.2 |
| 01.02 Nitrogen, sulfur and selenium metabolism | | | | | | | | |  |  |
| 01.02.02 Nitrogen metabolism | | | | | | | | |  |  |
| 60 | NAD(PH) nitroreductase | 111 | Y587_XANC5 |  | 21.0/5.83 | 20.0/4.6 | 7/31% | -5.6 | 28.8±7.4 | 161.9±14.3 |
| 01.05 C-compounds and carbohydrate metabolism | | | | | | | | |  |  |
| 220 | UDP-glucose dehydrogenase | 125 | Q8PGN5_XANAC |  | 43.1/6.18 | 68.0/6.7 | 13/25% | +2.6 | 141.3±24.1 | 54.9±16.8 |
| 01.06 Lipid, fatty acid and isoprenoid metabolism | | | | | | | | |  |  |
| 01.06.02 Membrane lipid metabolism | | | | | | | | |  |  |
| 38b | Outer membrane protein (FadL) | 156 | Q8PRE4_XANAC |  | 47.3/5.18 | 48.0/6.1 | 13/18% | -1.8 | 87.8±4.6 | 158±16.2 |
| 609 | Outer membrane protein (FadL) | 1070 | Q8PRE4_XANAC |  | 47.3/5.18 | 54.0/6.0 | 54/40% | +2.6 | 136.4±13.8 | 52.7±9.7 |
| 01.20 Secondary metabolism | | | | | | | | |  |  |
| 434 | Short chain dehydrogenase | 141 | Q8PME5_XANAC |  | 26.0/5.97 | 29.0/4.5 | 14/34% | -5.1 | 42.0±7.3 | 213.3±12.6 |
| 533 | Coproporphyinogen-III oxidase, aerobic | 191 | HEM6_XANAC |  | 34.6/5.81 | 48.0/5.4 | 11/30% | -1.5 | 61.2±4.5 | 91.9±4.9 |
| 02 Energy | | | | | | | | |  |  |
| 02.04 Glyoxylate cycle | | | | | | | | |  |  |
| 331 | KDPG and KHG aldolase | 163 | Q8PKU5_XANAC |  | 22.9/5.24 | 23.0/4.8 | 7/31% | -2.0 | 77.2±26.1 | 155.3±11.8 |
| 02.10 Tricarboxylic-acid pathway | | | | | | | | |  |  |
| 98a | Malate dehydrogenase | 905 | MDH_XANAC |  | 34.9/5.37 | 48.0/4.3 | 46/51% | +1.5 | 89.1±4.6 | 61.1±15.8 |
| 121 | Dihydrolipoamide S-succinyltransferase | 136 | Q3BVA5_XANC5 |  | 42.4/5.87 | 69.0/6.5 | 9/10% | +1.8 | 91.7±7.7 | 51.4±11.9 |
| 235 | Citrate synthase | 218 | Q3BPS8_XANC5 |  | 47.9/5.97 | 68.0/6.6 | 8/20% | +2.6 | 81.0±5.9 | 31.4±7.5 |
| 591a | Succinate dehydrogenase flavoprotein subunit | 206 | Q3BTD_XANC5 |  | 65.8/5.89 | 55.0/4.4 | 18/22% | -7.4 | 34.8±8.1 | 257.5±15.7 |
| 02.45 Energy conversion and regeneration | | | | | | | | |  |  |
| 02.45.15 Energy generation | | | | | | | | |  |  |
| 442a | Phosphoglycerate kinase | 688 | AAM38190 |  | 40.9/4.91 | 45.0/6.0 | 47/43% | -1.9 | 72.2±8.9 | 140.3±8.8 |
| 422 | NADH-ubiquinone oxidoreductase NQO1 subunit | 40 | Q3BRN4_XANC5 |  | 48.8/6.32 | 33.0/4.4 | 8/18% | -3.9 | 53.2±5.3 | 208.9±18.1 |
| 76 | ATP synthase beta chain | 72 | Q2P7Q4_XANOM |  | 51.0/5.18 | 32.0/6.1 | 3/8% | -10.7 | 67.0±26.9 | 715.7±42.4 |
| 11 Transcription | | | | | | | | |  |  |
| 11.04 RNA processing | | | | | | | | |  |  |
| 153a | Polyribonucleotide phosphorylase | 137 | PNP_XANAC |  | 75.5/5.47 | 28.0/5.9 | 6/3% | -1.5 | 87.3±4.9 | 133.1±11.1 |
| 12 Protein synthesis | | | | | | | | |  |  |
| 12.01 Ribosome biogenesis | | | | | | | | |  |  |
| 79 | 50S ribosomal protein L4 | 133 | AAM35856 |  | 21.8/9.68 | 14.0/5.9 | 4/15% | +5.1 | 151.8±11.7 | 30±9.8 |
| 331b | 50S ribosomal protein L4 | 85 | RL4_XANOR |  | 21.7/9.68 | 23.0/4.9 | 1/10% | -2.0 | 77.2±26.1 | 155.3±11.8 |
| 12.04 Translation | | | | | | | | |  |  |
| 26 | Elongation factor Tu | 294 | Q3BWY6_XANC5 |  | 43.3/5.45 | 67.0/6.2 | 25/24% | +2.2 | 97.7±5.9 | 45.2±13.9 |
| 173 | Elongation factor Tu | 329 | Q3BWY6_XANC5 |  | 43.3/5.45 | 48.0/5.9 | 20/42% | +4.4 | 54.2±6.9 | 238.7±19.5 |
| 153b | Methionyl-tRNA synthetase | 119 | SYM_XANAC |  | 75.9/5.72 | 28.0/5.10 | 16/10% | -1.5 | 87.3±4.9 | 133.1±11.1 |
| 14 Protein fate (folding, modification and destination) | | | | | | | | |  |  |
| 14.01 Protein folding and stabilization | | | | | | | | |  |  |
| 416 | Chaperone protein | 98 | DNAK_XANOM |  | 68.9/5.02 | 66.0/6.3 | 10/12% | +2.9 | 196.5±26.3 | 67.2±22.3 |
| 452 | 60 kDa Chaperonin | 71 | CH60_XANAC |  | 57.1/5.05 | 48.0/5.7 | 5/8% | -3.7 | 54.1±3.8 | 198.1±16.4 |
| 20 Cellular transport, transport facilities and transport routes | | | | | | | | |  |  |
| 20.03 Transport facilities | | | | | | | | |  |  |
| 151b | Regulator of pathogenicity factors | 104 | Q8PJM6_XANAC |  | 41.3/5.98 | 41.0/4.3 | 8/21% | +3.2 | 184.7±25.2 | 57.5±5.6 |
| 429 | Regulator of pathogenecity factors | 729 | Q8PJM6_XANAC |  | 41.3/5.98 | 47.0/4.5 | 55/61% | +2.7 | 184.4±30.7 | 67.4±14.5 |
| 477 | Outer membrane efflux protein RaxC precursor | 39 | Q3BPJ1_XANC5 |  | 49.6/5.46 | 27.0/6.1 | 2/3% | -3.7 | 48.7±3.8 | 178.2±6.1 |
| 486 | Regulator of pathogenicity factors | 231 | Q8PJM6_XANAC |  | 41.3/5.98 | 48.0/5.2 | 16/30% | +2.2 | 131.2±14.6 | 59.2±15.4 |
| 526 | Carbohydrate-selective porin | 183 | Q3BS50_XANC5 |  | 46.4/7.10 | 48.0/5.3 | 16/21% | +1.8 | 127.4±8.8 | 69.5±12.4 |
| 555 | Regulator of pathogenicity factors | 179 | Q8PJM6_XANAC |  | 41.3/5.98 | 42.0/4.8 | 16/18% | +2.8 | 127.7±10.5 | 45.4±14.8 |
| 20.09 Transport routes | | | | | | | | |  |  |
| 20.09.16 Cellular export and secretion | | | | | | | | |  |  |
| 49a | Putative secreted protein precursor | 163 | Q3BZA9_XANC5 |  | 24.5/9.18 | 18.0/5.8 | 7/20% | -2.4 | 73.3±5.3 | 175.4±20.9 |
| 30 Cellular communication/Signal transduction mechanism | | | | | | | | |  |  |
| 103a | OmpA-related protein | 371 | Q8PER6_XANAC |  | 110.1/5.29 | 75.0/5.9 | 28/16% | +2.9 | 113.1±4.6 | 39.1±8.5 |
| 1 | TonB-dependent receptor | 1406 | Q8PI48_XANAC |  | 105.8/4.76 | 42.0/4.1 | 89/34% | +2.9 | 131.6±23.4 | 44.8±8.6 |
| 2 | TonB-dependent receptor | 1441 | Q8PI48_XANAC |  | 105.8/4.76 | 58.0/6.7 | 85/35% | +2.9 | 147.7±19.7 | 50.1±10.4 |
| 15 | TonB-dependent receptor | 229 | Q8PH16_XANAC |  | 103.2/4.79 | 66.0/6.4 | 20/14% | -3.5 | 50.7±5.8 | 177.6±15.1 |
| 74 | TonB-dependent receptor | 597 | Q8PI48_XANAC |  | 105.8/4.76 | 20.0/4.7 | 27/15% | +3.4 | 178.0±10.2 | 52.7±8.5 |
| 168a | TonB-dependent receptor | 636 | Q8PGX3_XANAC |  | 89.0/5.00 | 55.0/6.0 | 38/29% | +4.9 | 277.7±99.9 | 56.3±13.5 |
| 168b | TonB-dependent receptor | 137 | Q8PIR5_XANAC |  | 86.3/4.97 | 55.0/6.2 | 16/10% | +4.9 | 277.7±99.9 | 56.3±13.5 |
| 219a | TonB-dependent receptor | 356 | Q8PI48_XANAC |  | 105.8/4.76 | 68.0/6.4 | 23/23% | +2.2 | 110.1±15 | 50.8±15.9 |
| 466 | TonB-dependent receptor | 113 | Q8PI27_XANAC |  | 97.3/5.14 | 54.0/6.8 | 7/4% | +3.6 | 252.2±46.2 | 70.3±24.1 |
| 38a | Ferric enterobactin receptor | 594 | Q8PHT1_XANAC |  | 87.3/5.20 | 48.0/6.0 | 44/21% | -1.8 | 87.8±4.6 | 158±16.2 |
| 55 | Outer membrane active sucrose transporter | 166 | Q2HPF0_9XANT |  | 88.9/4.93 | 58.0/6.4 | 8/9% | +2.8 | 173.6±31.2 | 61.2±3.6 |
| 219b | Outer membrane active sucrose transporter | 116 | Q2HPF0_9XANT |  | 88.9/4.93 | 68.0/6.5 | 4/6% | +2.2 | 110.1±15.1 | 50.8±15.9 |
| 30.01.05.01 Protein kinase | | | | | | | | |  |  |
| 49b | Adenylate kinase | 93 | Q3BPM9_XANC5 |  | 19.9/5.33 | 18.0/5.9 | 8/24% | -2.4 | 73.3±5.3 | 175.4±20.9 |
| 420 | Putative two-component system sensor histidine kinase | 40 | Q3BTZ4_XANC5 |  | 45.9/5.33 | 48.0/5.5 | 10/13% | -2.2 | 55.2±16.1 | 121.6±4.1 |
| 34 Interaction with the environment | | | | | | | | |  |  |
| 86 | YapH protein | 51 | Q8PKM0_XANAC |  | 306.9/4.15 | 68.0/5.5 | 1/0% | +4.1 | 178.1±41.1 | 43.8±15.1 |
| 42 Biogenesis of cellular components | | | | | | | | |  |  |
| 42.27 Extracellular/secretion protein | | | | | | | | |  |  |
| 103b | OmpA-related protein | 131 | Q8PFD5_XANAC |  | 109.3/5.21 | 75.0/5.10 | 6/5% | +2.9 | 113.1±4.6 | 39.1±8.5 |
| 432 | OmpW family outer membrane protein precursor | 151 | Q3BP00_XANC5 |  | 23.8/4.97 | 17.0/6.1 | 5/13% | +2.2 | 120.9±10.5 | 54.9±11.1 |
| 151a | Aromatic-amino-acid aminotransferase | 123 | Q2P9K5_XANOM |  | 43.3/5.82 | 41.0/4.2 | 4/9% | +3.2 | 184.7±25.2 | 57.5±5.6 |
| 168c | Oar protein | 261 | Q8PJ02_XANAC |  | 111.7/5.18 | 55.0/6.1 | 13/9% | +4.9 | 277.7±99.9 | 56.3±13.5 |
| 99 Unclassified proteins | | | | | | | | |  |  |
| 442b | AE011680 NID | 437 | AAM35431 |  | 57.1/5.05 | 45.0/6.1 | 25/26% | -1.9 | 72.2±8.9 | 140.3±8.8 |
| 591b | AM039952 NID | 97 | XAJ22623 |  | 155.6/7.82 | 55.0/4.5 | 8/5% | -7.4 | 34.8±8.1 | 257.5±15.7 |
| 188 | Putative secreted protein precursor | 792 | Q3BP07_XANC5 |  | 29.9/4.64 | 26.0/6.7 | 55/47% | +1.6 | 100.1±3.4 | 62.5±9.1 |

^a^ Fold change in biofilm compared to planktonic cultures

^b, c^ Average normalized spot volumes and standard deviations in biofilm and planktonic cultures, respectively
