# Supplementary material for: Insights into xanthomonas axonopodis pv. citri biofilm through proteomics
Source: BMC Microbiol. 2013 Aug 7;13:186. doi: 10.1186/1471-2180-13-186 (PMC3750573; doi:10.1186/1471-2180-13-186)
Supplement: Additional file 2: Table S2 — Oligonucleotides used in qRT-PCR of selected genes. [file 1471-2180-13-186-S2.doc]

**Supplementary Table 2. Oligonucleotides used in qRT-PCR of selected genes**

| **Protein** | **Gene name** | **Primer forward** | **Primer reverse** |
| --- | --- | --- | --- |
| UDP-glucose dehydrogenase | XAC3581 | ACATCGTCGAGTCCAAGGTC | CGACCCGGTGTTGAAATAGT |
| 50S ribosomal protein L4 | XAC0973 | GAAGGTCAACCGCAAGATGT | AGCGGACAGATACAGGTGCT |
| Elongation factor Tu (EfTu) | XAC0957 | GCTACCGTCCGCAGTTCTAC | CTTGATGATCTTGGCAACCA |
| Regulator of pathogenicity factor (RpfN) | XAC2504 | GAACAGCAGGTGACCAATCC | ATCGCTTCGTCGCTGTATTT |
| TonB-dependent receptor | XAC3489 | TGAAGGGTTTACCCTCAACG | CTTCAACTGATTGGCGTCCT |
| YapH | XAC2151 | AGTTCACCAACGTCGTTTCC | CTGATAGATCGGGCTGGTGT |
| Outer membrane protein OmpW | XAC3664 | CACCTCCTCGACTTCTTCCA | GCCCAGTTGTCGTTGATGTA |
| Molecular charperone DnaK | XAC1522 | GCTCTGTCGGATCTGGAAAC | TCCTTGACCTCGGTGAACTC |
|  | 16S | TGGTAGTCCACGCCCTAAACG | CTGGAAAGTTCCGTGGATGTC |
